# Improved RNA toehold switch sensitivity using the NanoLuc complementation reporter

**DOI:** 10.17912/micropub.biology.001334

**Published:** 2025-01-02

**Authors:** Katherinne J Diaz, Julian Jarquin, Aleksandr Petrosyan, Melissa K Takahashi

**Affiliations:** 1 Biology Department, California State University, Northridge, Northridge, California, United States

## Abstract

RNA toehold switches are powerful tools that can be used as biosensors to detect nearly any RNA sequence. In the presence of a specific RNA trigger, the toehold switch allows translation of a reporter protein. Toehold switches expressed in cell-free expression systems have been used as biosensors for several viruses and bacterial RNAs. Here we explore the use of the NanoLuc complementation reporter NanoBiT and found that it provides improved sensitivity over sfGFP. This reporter can be applied broadly across other RNA toehold switch applications.

**Figure 1. NanoBiT reporter improves RNA toehold switch sensitivity in cell-free reactions:**
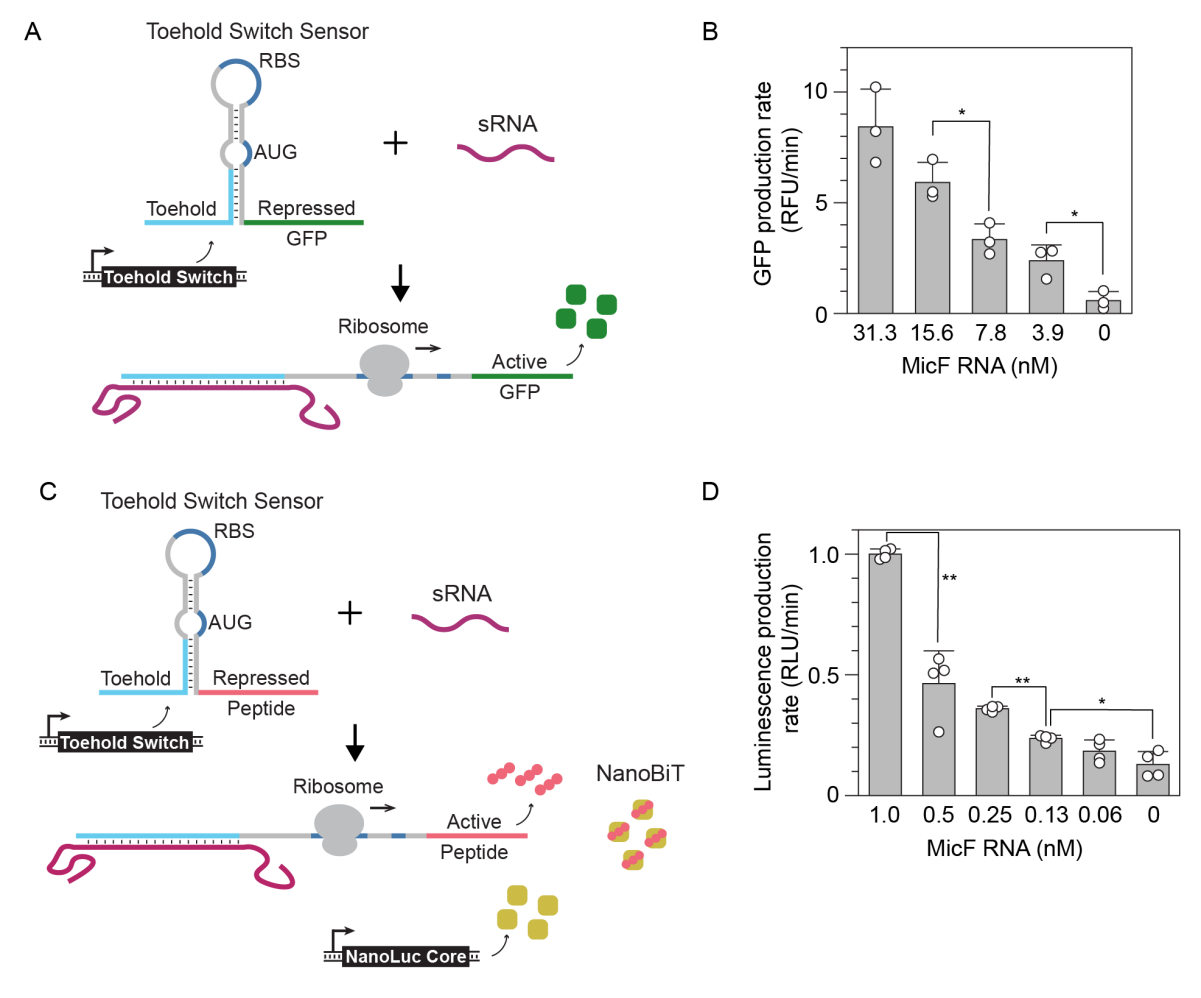
A) Schematic of RNA toehold switch mechanism. The toehold switch is constitutively transcribed in the cell-free reaction and controls translation of sfGFP. In the presence of the sRNA MicF, the reporter protein is translated. B) Average GFP production rate from CFE reactions with varying concentrations of MicF RNA taken at 25 minutes. Error bars represent standard deviation of three replicates depicted with open circles. C) Schematic of toehold switch controlling the translation of the complementation peptide. A separate DNA construct constitutively expresses the NanoLuc core protein. In the presence of the sRNA MicF, the complementation peptide is translated and is allowed to bind to the core protein forming the NanoBiT reporter. D) Average luminescence production rate from CFE reactions with varying concentrations of MicF RNA taken at 17.5 minutes. Error bars represent standard deviation of four replicates depicted with open circles. Replicates were collected from two independent experiments. T-tests were performed between adjacent concentrations in B and D. A Bonferroni correction was applied for multiple comparisons (* p<0.05, ** p < 0.001).

## Description

RNA toehold switches are synthetic riboregulators that can be designed to detect nearly any RNA sequence (Green et al., 2014) . When transcribed, the toehold switch folds into a secondary structure that prevents translation of the downstream mRNA by occluding the ribosome binding site (RBS) and start codon. Binding of a “trigger” RNA to the toehold region of the switch unwinds the secondary structure, allowing translation of the downstream gene. These riboregulators are powerful tools especially when combined with a cell-free expression (CFE) system which creates an *in vitro* biosensor. Toehold switch biosensors have been developed for several viruses including Ebola (Pardee et al., 2014) , Zika (Pardee et al., 2016) , Norovirus (Ma et al., 2018) , and SARS-CoV2 (Hunt et al., 2022; Park & Lee, 2021) . A limitation of the toehold switch is sensitivity; thus it is often paired with an isothermal amplification method to detect clinically relevant concentrations of RNA (Ma et al., 2018; Pardee et al., 2016; Park & Lee, 2021) . Another potential application for toehold switches is in detection of shorter RNAs such as microRNAs (miRNA) (McSweeney et al., 2023) and bacterial small RNAs (sRNA) (Green et al., 2014) . Due to the short length of these RNAs it would be preferable to directly detect the RNAs with the toehold switch without the need for amplification. To address this, we explored using different reporter proteins to improve sensitivity.

We designed a toehold switch to detect the sRNA MicF (see Methods). The two reporter proteins tested were sfGFP (Pédelacq et al., 2006) and the NanoLuc complementation reporter NanoBiT (Dixon et al., 2016) . For the complementation reporter, translation of the 11 amino acid peptide was controlled by the toehold switch while the core protein was constitutively expressed in the CFE reaction. Toehold switches were tested by providing varying concentrations of *in vitro* transcribed MicF RNA in each reaction. As seen in Figure 1, the 7.8 and 3.9 nM RNA concentrations were not statistically different using the sfGFP reporter. Whereas the 0.25 and 0.13 nM RNA concentrations were distinguishable using the NanoBiT reporter. Further testing is required to determine the limit of detection using these reporters. However, initial testing suggests that the NanoBiT reporter does provide improved sensitivity over sfGFP and could have widespread use in toehold switch biosensor applications.

## Methods

RNA toehold switch design and cloning

Toehold switch sensors were designed with NUPACK (Zadeh et al., 2011) using the series B toehold switch design from Pardee et al. (Pardee et al., 2016) . NUPACK code can be found in Wu et al. (Wu et al., 2022) . Toehold switch sensors were cloned into plasmids with either superfolder green fluorescent protein (sfGFP) (Pédelacq et al., 2006) or NanoBiT (Dixon et al., 2016) reporters using PCR amplification and blunt-end ligation. Plasmids used in cell-free reactions were purified using the QIAfilter Plasmid Midi Kit (Qiagen 12243) followed by isopropanol precipitation and eluted with nuclease-free water. Plasmids can be found at Addgene (Table 1). Key sequences can be found in Table 2.

In vitro RNA synthesis

MicF RNA was in vitro transcribed using the HiScribe T7 Quick High Yield RNA Synthesis Kit (New England Biolabs E2050S) according to manufacturer’s instructions. RNA was purified using the RNA Clean and Concentrator-25 Kit (Zymo Research R1017) and eluted with nuclease-free water.

Cell-free extract and buffer preparation

Cell extract was prepared according to Didovyk et al. (Didovyk et al., 2017) with the following modifications. A single colony of *E. coli* BL21 Star (ThermoFisher Scientific C601003) transformed with the pAD-LyseR plasmid (Didovyk et al., 2017) was grown overnight at 37°C while shaking in lysogeny broth (LB) with carbenicillin (100 μg/ml). The production culture was grown in 2xYTPG medium (400 μl of overnight culture added to 400 ml of 2xYTPG) at 37°C until an OD _600_ of 1.5 was reached. Cells were harvested by centrifugation at 1800g for 15 min at room temperature. The supernatant was discarded, and the pellet was resuspended with 45 ml of cold S30A buffer. The resuspended cells were centrifuged again in a pre-weighed conical tube at the same settings as above. The supernatant was discarded, and the pellet was weighed. The pellet was resuspended in two volumes relative to pellet weight (e.g. 2.6 ml for a 1.3 g pellet) of cold S30A buffer supplemented with 2 mM dithiothreitol (DTT) and frozen at -80°C. The frozen pellet was thawed at room temperature in a water bath then vortexed for two to three minutes. The thawed pellet was then incubated while shaking at 37°C for 45 minutes. Vortexing and incubation was repeated. The extract was distributed into microcentrifuge tubes rated for 30,000g and centrifuged at 30,000g for 45 minutes at 4°C. The supernatant was collected and dialyzed in S30B buffer supplemented with 1 mM DTT. Dialysis was performed according to Slide-a-lyzer (Thermo Scientific 66380) instructions for 3 hours at 4°C. The dialyzed extract was centrifuged at 21,000g for 5 minutes to remove any remaining cell debris. Aliquots (35 ul each) of the extract were frozen at -80C.

The reaction buffer and energy mix was prepared according to Sun et al. (Sun et al., 2013) . Additional magnesium glutamate was optimized per extract batch as described previously (Didovyk et al., 2017; Sun et al., 2013) . A typical 10 μl reaction included 4 μl of extract, 0.71 μl of 14x energy mix, 3.5 μl of buffer, and 0.25 μl of SUPERase-In RNase inhibitor (ThermoFisher Scientific AM2694). The remainder of the volume was used to add the desired DNA or RNA constructs. The buffer volume would be adjusted for the optimized magnesium glutamate concentration. Reactions using the NanoBiT reporter utilized the Nano-Glo Luciferase Assay System (Promega N1120) according to the manufacturer recommended concentrations.

2xYTP: 18.6 g 2YT (BD 244020), 1.8 g potassium phosphate monobasic (F.W. 136.09), 4.2 g potassium phosphate dibasic (F.W. 174.18), fill to 600 ml with DI water, autoclave sterilize. 2xYTPG: Add 7.2 g D-glucose (F.W. 180.16) to 400 ml of 2xYTP, filter sterilize. S30A: 50 mM Tris-HCl (pH 7.7), 60 mM potassium glutamate, 14 mM magnesium glutamate, adjust to pH 7.7. S30B: 60 mM potassium glutamate, 14 mM magnesium glutamate, use 2 M Tris base to adjust pH to 8.2.

Cell-free experiment and data analysis

sfGFP reporter : 1 nM of toehold switch sensor plasmid was added to each reaction along with the MicF RNA concentration listed in the figure. Cell-free extract, reaction buffer, energy mix, and RNase inhibitor were mixed together and incubated at 37°C for 20 minutes. Upon incubation the cell-free reaction mixture was added to PCR tubes containing the desired DNA and RNA constructs on ice. Reactions were vortexed and then 8.5 μl were distributed to wells on a 384-well microtiter plate (Thermo Scientific 242764) in triplicate. The microtiter plate was sealed with a clear seal (VWR 60941-078) and incubated at 37°C on a Biotek Synergy H1m microtiter plate reader for 2 hours. Fluorescence measurements were taken every 5 minutes with excitation 485 nm and emission 520 nm. The starting fluorescence for each reaction was adjusted to zero by subtracting the time zero fluorescence from each of the time points. The average fluorescence from the control wells without any DNA or RNA was subtracted from each reaction condition. A fluorescence production rate was calculated at each time point: (t _2_ – t _1_ )/5. A moving average of the production rate was calculated across three time points. The average of the moving average at 25 minutes was reported in the figure. Where 25 minutes corresponds to t _1_ .

NanoBiT reporter: 1 nM of the NanoLuc core plasmid and 0.25 nM of the toehold switch sensor plasmid was added to each reaction along with the MicF RNA concentration listed in the figure. Cell-free extract, reaction buffer, energy mix, and RNase inhibitor were mixed together and incubated at 37°C for 20 minutes. Upon incubation the cell-free reaction mixture was placed on ice and 0.2 μl of Nano-Glo reagent was added per 10 μl of reaction volume. The final mixture was added to PCR tubes containing the desired DNA and RNA constructs on ice. Reactions were vortexed and then 9 μl were distributed to wells on a 384-well microtiter plate. The microtiter plate was sealed with a clear seal and incubated at 37°C on a Biotek Synergy H1m microtiter plate reader for 1 hour. Luminescence measurements were taken every 2.5 minutes. The average luminescence from the control wells without any DNA or RNA was subtracted from each reaction condition. A luminescence production rate was calculated at each time point: (t _2_ – t _1_ )/2.5. A moving average of the production rate was calculated across three time points. The average of the moving average at 17.5 minutes was reported in the figure. Where 17.5 minutes corresponds to t _1_ .

## Reagents

Table 1. Plasmids used in this study.

**Table:** 

Name	Description	Origin/resistance	Addgene ID
MKT071	J23119-MicF toehold switch-linker-sfGFP	ColE1/Kan	231995
MKT073	J23119-RBS-Nluc-core	ColE1/Kan	231996
MKT074	J23119-MicF toehold switch-linker-P86 Nluc comp peptide	ColE1/Kan	231997

Table 2. Key DNA sequences

**Table:** 

Name	Sequence (5'-to-3')
MicF toehold switch	GTGAATGACGGTAATAAATAAAGTTAATGATGATAGCGGACTTTAGAACAGAGGAGATAAAGATGGCTATCATCATGGGAGGT TCTGGAGGT
P86 NanoLuc complementation peptide	GTCAGCGGATGGCGACTCTTCAAGAAGATCAGC
11S NanoLuc core protein	GTGTTTACCCTGGAAGATTTTGTGGGCGATTGGGAACAGACCGCGGCGTATAACCTGGATCAGGTGCTGGAACAGGGCGGCG TGAGCAGCCTGCTGCAGAACCTGGCGGTGAGCGTGACCCCGATTCAGCGCATTGTGCGCAGCGGCGAAAACGCGCTGAAAAT TGATATTCATGTGATTATTCCGTATGAAGGCCTGAGCGCGGATCAGATGGCGCAGATTGAAGAAGTGTTTAAAGTGGTGTATCC GGTGGATGATCATCATTTTAAAGTGATTCTGCCGTATGGCACCCTGGTGATTGATGGCGTGACCCCGAACATGCTGAACTATTTT GGCCGCCCGTATGAAGGCATTGCGGTGTTTGATGGCAAAAAAATTACCGTGACCGGCACCCTGTGGAACGGCAACAAAATTAT TGATGAACGCCTGATTACCCCGGATGGCAGCATGCTGTTTCGCGTGACCATTAACAGC
